# Editorial: Physical Fitness and Cardiovascular Health in Specific Populations

**DOI:** 10.3389/fcvm.2022.874874

**Published:** 2022-04-19

**Authors:** Gen-Min Lin, Chih-Lu Han

**Affiliations:** ^1^Department of Internal Medicine, Hualien Armed Forces General Hospital, Hualien, Taiwan; ^2^Department of Internal Medicine, Tri-Service General Hospital and National Defense Medical Center, Taipei, Taiwan; ^3^Department of Internal Medicine, Taipei Veterans General Hospital, Taipei, Taiwan

**Keywords:** athlete's heart, physical activity, physical fitness, predictive models, specific populations, sex difference, myocarditis

Athlete's heart has been regarded as a benign phenotype of cardiac remodeling related to physical training. Several electrocardiographic changes such as first-degree atrioventricular block and left ventricular hypertrophy are prevalent in athletes (1). In cardiac imaging studies, athletes have greater chamber sizes and more effective diastolic function than sedentary individuals (2). Previous studies have revealed racial differences in the cardiac adaptions to exercise and to left ventricular pressure overload. For given levels of training, athletes of African/Afro-Caribbean descent demonstrate more marked morphologic changes than Caucasian athletes, which is likely due in part to genetic factors (3, 4). In addition, the cardiac remodeling in male athletes is greater than that in female athletes Wooten et al.. However, there have only a few studies investigating cardiovascular health in Asian athletes and in some specific subgroups such like military individuals who have to receive regular training but are at high risk of psychological stress and metabolic abnormalities. In the current Topic Research, we have collected 9 high quality studies for investigating cardiovascular health and prognosis in athletes and specific populations.

From an Asian population of physically active military personnel in Taiwan (5–7), Liu et al. demonstrated that cardiac structural and functional characteristics differ between endurance and strength elite male athletes. While greater left ventricular mass index predicts elite status in both groups of male athletes, consistent with findings from Western elite athletes, greater diastolic function and right ventricular systolic pressure characterize strength elite athletes, while lower heart rate at rest predicts endurance elite athletic status. Lin et al. further found a sex difference that only greater right ventricular chamber size could characterize elite female strength athletes. For the electrocardiographic (ECG) changes in physically active obese military males, Lin et al. uncovered that obesity which was defined as body mass index ≥27.0 kg/m^2^ was associated with higher risk of ECG based left atrial enlargement and T wave inversion in inferior leads, whereas the risk between obesity and ECG based left ventricular hypertrophy might vary by the ECG criteria, possibly due to a high prevalence of exercise induced-left ventricular hypertrophy in military and greater chest wall thickness in obesity. This study highlighted that the cardiac prognosis for various ECG criteria defined left ventricular hypertrophy in physically active obese adults requires further investigation.

In a Korean population of 68,223 individuals older than 65 years of age, Kim et al. displayed that compared with the sedentary group, the physically active groups with and without cardiovascular diseases (CVD) had a lower risk of all-cause death with a median follow-up of 42 months. A 500 metabolic equivalent task-minute per week increase in physical activity resulted in an 11% and 16% reduction in the mortality risk in the non-CVD and CVD groups, respectively. Kim et al. found that in the elderly, the benefits of physically activity in patients with CVD, especially stroke or heart failure, were greater than those without. In another Chinese population of 2,830 individuals older than 65 years of age, Lyu et al. showed that after adjusting for CVD risk factors, there was a negative association of weekly walking activity with vascular hypertensive mediated organ damage (HMOD), objectively evaluated by carotid-femoral pulse wave velocity, carotid intima–media thickness, and ankle-brachial index. Increased daily walking duration ≥1 h, but not walking frequency, was significantly associated with improved vascular HMOD in the elderly Chinese. In a British population of 5,300 individuals without CVD who had an average of 68 years of age, Liu et al. demonstrated that the low grip strength trajectory pattern was associated with higher risk of incident CVD for a median of 6.1-year follow-up. This study emphasized that continuous measurement of grip strength values could help identify the elderly individuals at risk of CVD.

For snorers with uncontrolled hypertension, Wang et al. developed a predictive model for the occurrence of coronary heart disease within 8 years in which the area under the receiver operating characteristic curve was 0.71. A total of more than 134 points in the nomogram can be used in the identification of high-risk patients for coronary heart disease in snorers with uncontrolled hypertension. In another paper developing a novel machine learning model to predict cardio-respiratory fitness level based on the anthropometric parameters and workload and steady-state heart rate of a submaximal exercise test, Xiang et al. found that the accuracy was 75%, and *R*^2^ in the groups of age 21–40 years and above age 40 years were 0.85 and 0.75, respectively, when the support vector machine was used. Finally, Wang et al. analyzed the statistics regarding the global, regional, and national burden of myocarditis from 1990 to 2017, which could provide a platform for further investigation into the myocarditis burden in the COVID-19 pandemic era.

Taken together, the present Research Topic represents an important source of up-to-date information, covering many aspects of physical activity, fitness and cardiovascular health in specific populations. More comprehensive knowledge according to these discoveries may bring about new perspectives.

## Author Contributions

All authors listed have made an equal, substantial, direct, and intellectual contribution to the work and approved it for publication.

## Conflict of Interest

The authors declare that the research was conducted in the absence of any commercial or financial relationships that could be construed as a potential conflict of interest.

## Publisher's Note

All claims expressed in this article are solely those of the authors and do not necessarily represent those of their affiliated organizations, or those of the publisher, the editors and the reviewers. Any product that may be evaluated in this article, or claim that may be made by its manufacturer, is not guaranteed or endorsed by the publisher.
